# Identification of 3,3′-*O*-dimethylellagic acid and apigenin as the main antiplasmodial constituents of *Endodesmia calophylloides* Benth and *Hymenostegia afzelii* (Oliver.) Harms

**DOI:** 10.1186/s12906-021-03352-9

**Published:** 2021-06-29

**Authors:** Rodrigue Keumoe, Jean Garba Koffi, Darline Dize, Patrick Valère Tsouh Fokou, Joseph Tchamgoue, Lawrence Ayong, Bruno Lenta Ndjakou, Norbert Sewald, Bathelemy Ngameni, Fabrice Fekam Boyom

**Affiliations:** 1 Antimicrobial and Biocontrol Agents Unit (AmBcAU), Laboratory for Phytobiochemistry and Medicinal Plants Studies, Department of Biochemistry, Faculty of Science, University of Yaoundé I, P.O. Box 812, Yaoundé, Cameroon; 2 Malaria Research Unit, Centre Pasteur du Cameroun, P.O. Box 1274, Yaoundé, Cameroon; 3 Higher Teachers Training College, University of Yaoundé I, P.O Box 47, Yaounde, Cameroon; 4 Organic and Bioorganic Chemistry, Faculty of Chemistry, Bielefeld University, D-33501 Bielefeld, Germany; 5 Laboratory of Pharmacognosy and Pharmaceutical Chemistry, Faculty of Medicine and Biomedical Sciences, University of Yaounde I, P.O Box 1364, Yaounde, Cameroon

**Keywords:** 3,3′-*O*-dimethylellagic acid, Apigenin, Antiplasmodial activity, Bioguided fractionation

## Abstract

**Background:**

*Endodesmia calophylloides* and *Hymenostegia afzelii* belong to the Guttiferae and Caesalpiniaceae plant families with known uses in African ethno-medicine to treat malaria and several other diseases. This study aimed at identifying antiplasmodial natural products from selected crude extracts from *H. afzelii* and *E. calophylloides* and to assess their cytotoxicity.

**Methods:**

The extracts from *H. afzelii* and *E. calophylloides* were subjected to bioassay-guided fractionation to identify antiplasmodial compounds. The hydroethanol and methanol stem bark crude extracts, fractions and isolated compounds were assessed for antiplasmodial activity against the chloroquine-sensitive 3D7 and multi-drug resistant Dd2 strains of *Plasmodium falciparum* using the SYBR green I fluorescence-based microdilution assay. Cytotoxicity of active extracts, fractions and compounds was determined on African green monkey normal kidney *Vero* and murine macrophage *Raw* 264.7 cell lines using the Resazurin-based viability assay.

**Results:**

The hydroethanolic extract of *H. afzelii* stem bark (Hasb^HE^) and the methanolic extract of *E. calophylloides* stem bark (Ecsb^M^) exhibited the highest potency against both *Pf*3D7 (EC_50_ values of 3.32 ± 0.15 μg/mL and 7.40 ± 0.19 μg/mL, respectively) and *Pf*Dd2 (EC_50_ of 3.08 ± 0.21 μg/mL and 7.48 ± 0.07 μg/mL, respectively) strains. Both extracts showed high selectivity toward *Plasmodium* parasites (SI > 13). The biological activity-guided fractionation led to the identification of five compounds (Compounds 1–5) from Hasb^HE^ and one compound (Compound 6) from Ecsb^M^. Of these, Compound 1 corresponding to apigenin (EC_50_ *Pf*3D7, of 19.01 ± 0.72 μM and EC_50_ *Pf*Dd2 of 16.39 ± 0.52 μM), and Compound 6 corresponding to 3,3′-*O*-dimethylellagic acid (EC_50_ *Pf*3D7 of 4.27 ± 0.05 μM and EC_50_ *Pf*Dd2 of 1.36 ± 0.47 μM) displayed the highest antiplasmodial activities. Interestingly, both compounds exhibited negligible cytotoxicity against both *Vero* and *Raw* 264.7 cell lines with selectivity indices greater than 9.

**Conclusions:**

This study led to the identification of two potent antiplasmodial natural compounds, 3,3′-*O*-dimethylellagic acid and apigenin that could serve as starting points for further antimalarial drug discovery.

**Supplementary Information:**

The online version contains supplementary material available at 10.1186/s12906-021-03352-9.

## Background

Malaria is a mosquito-borne disease caused by protozoan parasites belonging to the genus *Plasmodium*. At least five *Plasmodium* species are known to cause malaria in humans, the deadliest of which is *Plasmodium falciparum*. Despite substantial global efforts towards its eradication, malaria remains a major public health problem, particularly in sub-Saharan Africa. According to the World Health Organization (WHO), an estimated 228 million cases and 405,000 deaths due to malaria occurred in 2018, with the Sub-Saharan African regions accounting for over 94% of the global mortality [1].

In the absence of vaccines with operational utility against malaria, accurate diagnosis and effective treatment remain the best hope of averting severe complications of the disease. Several antimalarial drugs including mefloquine, chloroquine, quinine, proguanil, atovaquone, sulphadoxine-pyrimethamine and artemisinin were developed for this purpose. However, rapid development of *P. falciparum* parasites resistance to such excepting artemisinin appeared [2]. The WHO therefore recommended the artemisinin-based combination therapies (ACTs) as first line antimalarial treatment since 2006 [3]. In the ACTs, the fast and short acting artemisinin is combined with long acting partners to exert improved effect against malaria parasites [4, 5]. Nevertheless, the ACTs are currently facing increasing threat of widespread *P. falciparum* resistance as delayed parasite clearance has been reported in South East Asia and Africa [6–8]. In addition, drug toxicity and high cost are associated with compliance issues to limit access and completion of malaria treatment in high-burdened settings. Overall, several challenges exist in the chemotherapy of malaria, and include widespread resistance and the limited number of drug choices available to manage multidrug-resistant parasite strains. To address these challenges, the antimalarial drug discovery pipeline should be continuously flushed-in with novel chemical scaffolds having promising features as starting points for new drugs development to combat malaria. One of the approaches to achieve this goal is to investigate herbal medicines and their derived secondary metabolites.

Historically, plants have played remarkable roles in antimalarial drug discovery, and they continue to serve as principal sources of new antimalarial therapies. Of note, the antimalarials, quinine and artemisinin are illustrative examples isolated from the barks of *Cinchona* species and leaves of *Artemisia annua* L., respectively [9, 10]. It is of common knowledge that the historical ethnomedicinal practices while using these plants to cure malaria further guided the isolation procedures of their active principles (quinine and artemisinin). Indeed, the bark of the *Cinchona* tree native to South America provides a rich source of medicinal alkaloids. The first use of the bark in treating malaria is often attributed to Jesuit missionaries in seventeenth century in Peru, though the indigenous population used hot infusions of the bark much earlier to combat shivering in cold and damp conditions. In the case of *Artemisia annua* L., the Chinese medicine indicated that “one bunch of qinghao (dried aerial part of *Artemisia annua* L.) in two *sheng* of water was mashed and the juice administered to patients.” Given the vast floristic diversity of Cameroon which is widely explored by indigenous population for the treatment of various illnesses including malaria, *H. afzelii* and *E. calophylloides* were selected for this study based on their ethnobotanical importance and the gap in their scientific exploration.

*Hymenostegia afzelii* and *E. calophylloides* belong respectively to Caesalpiniaceae and Guttiferae plant families that are broadly distributed in Cameroon. Species from these plant families are widely used in African folk medicine for the treatment of several illnesses including malaria [11–14]. Of note, *H. afzelii* is used in African traditional medicine to treat various diseases. A decoction of leaves and roots is used in Ghana to treat cough and whooping cough and wounds. The twigs are used throughout west Africa as chewing sticks to clean teeth [11, 12, 15]. *Endodesmia calophylloides*, the sole species in the *Endodesmia* genus is also used in folk medicine to heal filariasis and diarrhea, and as eye-instillation [16, 17]. Though a single report has mentioned the antiplasmodial activity of extracts from *E. calophylloides* [18], the potential of *H. afzelii* extracts against malaria parasites remains to be documented. The present paper reports the bioassay-guided isolation of two antiplasmodial compounds, apigenin (1) and 3,3′-*O*-dimethylellagic acid (6) from *H. afzelii* and *E. calophylloides*, respectively. The in vitro selectivity profile of the antiplasmodial extracts, fractions and compounds versus normal mammalian cells is also reported as an indicator of their toxicity to parasitic but not to mammalian cells at an early stage in a screening procedure for further evaluation in animals.

## Methods

### General experimental procedures

The methanol and hydroethanol were used as solvents for the extraction of plant material; *n*-hexane, dichloromethane (CH_2_Cl_2_), ethyl acetate (EtOAc) and methanol (MeOH) were used as pure or dual mixtures at different polarities for isolation of compounds. The ^1^H and ^13^C NMR spectra were registered at 500 MHz and 125 MHz, respectively, on Bruker DRX 500 NMR spectrometers (Bruker, Rheinstetten, Germany), with tetramethylsilane as reference, giving the chemical shifts in ppm and the coupling constants in Hertz. Column chromatographies were carried out on 230–400 mesh silica gel (Merck, Darmstadt, Germany), 70–230 mesh silica gel (Merck, Darmstadt, Germany) and sephadex LH-20 (Sigma-Aldrich, Munich, Germany). Precoated plates of silica gel 60 F254 (Merck; Darmstadt, Germany) were used for analytical purposes and the spots were detected with a UV lamp at 254 and 366 nm and by spraying with 50% H_2_SO_4_ followed by heating.

### Plant material

The stem barks of *E. calophylloides* Benth. and *H. afzelii* (Oliv.) Harms were collected in the Center Region of Cameroon at Mbalmayo and Mount Kala, respectively and identified by Mr. Nana Victor, a retired botanist at the National Herbarium of Cameroon. The voucher specimens were kept under the reference numbers 29,528 /HNC and 45,345/HNC for *E. calophylloides* and *H. afzelii*, respectively.

### Parasite strains and cell lines

The in vitro antiplasmodial assay was performed using the chloroquine-sensitive 3D7 (MRA-102) and multi-resistant Dd2 (MRA-150) strains of *P. falciparum* obtained from Bei Resources (https://www.beiresources.org/). The cytotoxicity assay was performed using the African green monkey normal kidney Vero cells (ATCC CRL 1586) and murine macrophages *Raw* 264.7 cells (ATCC #TIB-71) obtained from the American Type Culture Collection (ATCC).

### Plant extraction and bio-guided fractionation

The plant samples were air-dried and ground into fine powder using an electric mill (Hammer Mill, Leabon 9FQ, Zhengzhou, PRC). The resulting powders (1.2 kg, *H. afzelii*; 3.0 kg, *E. calophylloides*) were extracted each by maceration three times at room temperature in a mixture of EtOH/H_2_O (7:3) and in methanol, respectively for 72 h. The resulting solutions were filtered, evaporated under vacuum and lyophilized in a freeze-dryer Alpha 2–4 LD plus (Christ, Germany) to yield four extracts viz. the hydroethanolic (234 g) and methanolic (150 g) extracts of *H. afzelii* and the hydroethanolic (222) and methanolic (246 g) extract of *E. calophylloides*. The yields of the large-scale extraction were calculated relative to the weight of the starting plant materials. The extracts were subjected to preliminary antiplasmodial screening and subsequently, bioassay-guided fractionation was performed with the affordable extracts.

**Fig. 1:**
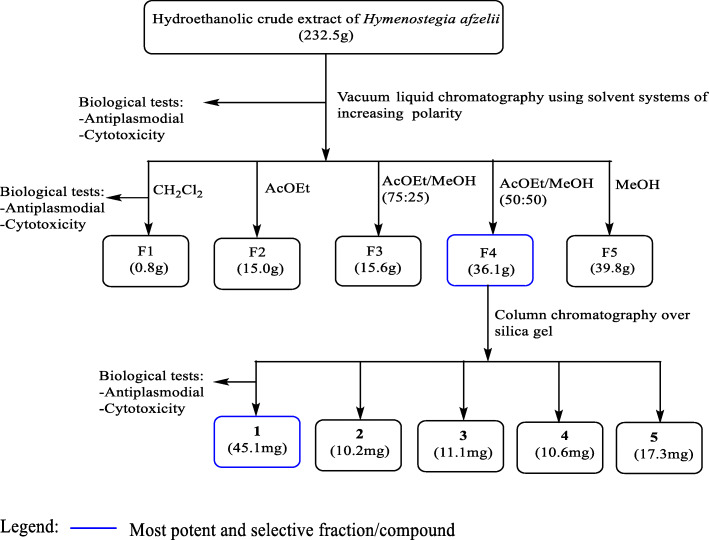
Bio-assay guided isolation of active compounds from the hydroethanolic crude extract of the stem bark of *H. afzelii*

Part of the hydroethanolic active crude extract (232.5 g) from stem bark of *H. afzelii* was subjected to vacuum liquid chromatography and successively eluted with CH_2_Cl_2_, EtOAc, mixtures of EtOAc/MeOH of increasing polarities and MeOH, leading to five fractions coded: F1 (0.8 g, CH_2_Cl_2_); F2 (15.0 g, EtOAc); F3 [15.6 g, EtOAc/MeOH (75:25), v/v]; F4 [36.1 g, EtOAc/MeOH (50:50), v/v] and F5 (39.8 g, MeOH). Among the promising fractions, F4 was afforded in higher quantity and demonstrated high antiplasmodial activity and higher selectivity (Table 2). Therefore, 35 g of F4 was further submitted to silica gel column chromatography using a stepwise gradient of EtOAc in *n*-hexane, then pure ethyl acetate followed by a gradient of MeOH in EtOAc to yield compounds 1 (45.1 mg, Hex/EtOAc 60:40, v/v), 2 (10.2 mg, Hex/EtOAc 50:50, v/v), 3 (11.1 mg, Hexane/EtOAc 40:60, v/v), 4 (10.6 mg, Hexane/EtOAc 50:50, v/v) and, 5 (17.3 mg, Hexane/EtOAc 95: 5, v / v) (Fig. 1).

**Fig. 2:**
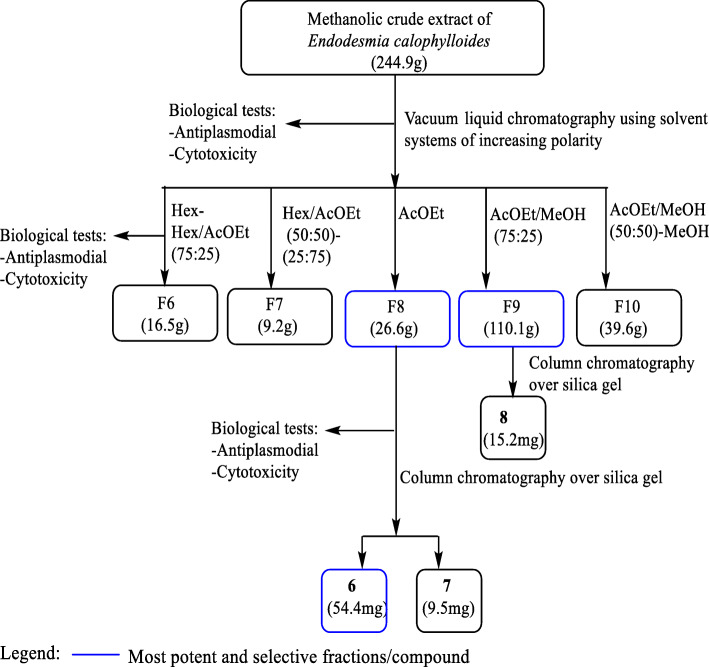
Bio-assay guided isolation of active compounds from the methanolic crude extract of the stem bark of *E. calophylloides*

Portion of the methanolic active crude extract (244.9 g) from the stem bark of *E. calophylloides* was subjected to a vacuum liquid chromatography and successively eluted with hexane, mixtures of Hex/EtOAc and EtOAc/MeOH of rising polarities and MeOH, leading to five fractions: F6 [16.5 g, Hex/EtOAc (75:25), v/v]; F7 [9.2 g, Hex/EtOAc (50:50–25:75), v/v]; F8 (26.6 g, EtOAc); F9 [110.1 g, EtOAc/MeOH (75:25), v/v] and F10 (39.6 g, MeOH). 25 g each of fractions F8 and F9 which demonstrated the best antiplasmodial activities (Table 2) were subjected to purification on an open column chromatography and eluted successively with a gradient of EtOAc in Hex and MeOH in EtOAc to afford compounds 6 (54.4 mg, Hex / EtOAc 30:70, v/v) and 7 (9.5 mg, EtOAc) from fraction F8, and compound 8 (15.0 mg, Hexane / EtOAC 20:80, v/v) from fraction F9 (Fig. 2).

### Antiplasmodial susceptibility testing

#### *Plasmodium falciparum* continuous culture and maintenance

The Chloroquine-sensitive 3D7 (MRA-102) and resistant *P. falciparum* Dd2 (MRA-150) strains were cultured in fresh O^+^ human red blood cells at 3% haematocrit in complete RPMI 1640 medium [500 mL RPMI 1640 (Gibco, UK) supplemented with 25 mM HEPES (Gibco, UK), 0.50% Albumax I (Gibco, USA), 1X hypoxanthine (Gibco, USA) and 20 μg/mL gentamicin (Gibco, China)] and incubating at 37 °C in a humidified atmosphere with 5% CO_2_. The culture medium was renewed daily to propagate the culture. Thin blood smears were made and stained with 10% Giemsa solution for 10 min and microscopically examined under oil immersion to monitor cell cycle transition and parasitaemia.

#### Synchronization of parasite culture

Before each experiment, synchronized ring stage parasites were obtained by 5% sorbitol (w/v) treatment as previously described [19]. The experiment with synchronized ring stage culture provided distinct observing growth inhibitory effect without a rise in parasitemia during the ring-trophozoite-schizont transitions.

#### SYBR green I fluorescence-based antiplasmodial assay

The parasite susceptibility was determined in 96-well microtitration plates using the SYBR green I fluorescence-based method [20] with some modifications. Briefly, sorbitol-synchronized ring stage parasites (hematocrit: 1.5%, parasitemia: 1%) were incubated in the presence of two-fold diluted extracts (0.78-100 μg/mL, DMSO 0.5%), fractions (0.39-50 μg/mL, DMSO 0.5%) or compounds (0.15-20 μg/mL, DMSO 0.5%). Artemisinin (98%, Sigma–Aldrich, Germany) and Chloroquine (98%, Sigma–Aldrich, Germany) were used as reference compounds at concentration ranges from 7.81–1000 nM. Drug-free culture wells in 0.5% DMSO were considered as positive growth controls. After 72 h of incubation, 50 μL of SYBR Green I lysis solution [Tris (20 mM; pH 7.5) (Sigma–Aldrich), EDTA (5 mM) (Sigma–Aldrich), saponin (0.008%, w/v) (Sigma–Aldrich), Triton X-100 (0.08%, v/v) (Sigma–Aldrich) and SYBR Green (2x) (Life technologies)] were added to each well and the plate was incubated in the dark at 37 °C for 30 min. SYBR green I fluorescence was measured using a Fluoroskan Ascent multi-well plate reader (Thermo scientific) with excitation and emission wavelength bands set at 485 and 538 nm, respectively. These data were normalized to percent control activity using Microsoft Excel software and median maximal effective concentrations (EC_50_s) calculated using GraphPad Prism 5.0 software (San Diego, California) with data fitted by nonlinear regression to the variable slope sigmoidal dose-response formula y = 100/[1+ 10^(logIC50-*x*)*H*^], where *H* is the hill coefficient or slope factor [21]. The resistance indices (RI), defined as the ratio of the EC_50_ of drug-resistant strain to the EC_50_ of sensitive strain were calculated.

### Cytotoxicity study

The cytotoxicity of active natural products was assessed according to the protocol described by [22]. The African green monkey normal kidney Vero cells (ATCC CRL 1586) and murine macrophages *Raw* 264.7 cells (ATCC #TIB-71) were maintained in T-25 flasks (Corning Incorporated, USA) using complete Dulbecco’s Modified Eagle’s Medium (Sigma-Aldrich, Germany), supplemented with, 10% Fetal Bovine Serum (Sigma-Aldrich, Germany), 0.2% sodium bicarbonate (w/v) (Sigma-Aldrich, Germany) and 1% (v/v) Penicillin-Streptomycin (Sigma-Aldrich, Germany). The cells were kept at 37 °C for 72 h in 5% CO_2_ incubator, and the medium was renewed each 72 h and the cell density monitored under the inverted fluorescent microscope Etaluma 520 (Etaluma, USA) until the formation of a monolayer. Confluent culture (nearly 90%) was trypsinized (0.05% Trypsin-EDTA, Sigma-Aldrich, Germany), then centrifuged at 1800 rpm for 5 min and the resulting pellet was re-suspended in culture medium. Cells at 10^4^cells per well were seeded (100 μL) in 96-well culture plates (Costar, USA) and incubated overnight to allow cell adhesion. Thereafter, 10 μL of serially diluted extracts, fractions (≤200 μg/mL), and compounds (≤50 μg/mL) were added to plate wells in duplicate. The plates were incubated in a humidified and 5% CO_2_ atmosphere at 37 °C for 48 h. Podophyllotoxin at 20 μM was added as positive control and wells containing untreated cells were included as 100% growth control. Ten microliters of resazurin stock solution (0.15 mg/mL in sterile PBS), were added to each well, and incubated for an additional 4 h. Fluorescence was then read using a Magelan Infinite M200 fluorescence multi-well plate reader (Tecan) with excitation and emission wavelengths at 530 and 590 nm, respectively. The percentage of cell viability was calculated with regard to the negative control, and subsequently used to determine the concentration that reduced 50% of cell viability (CC_50_) by non-linear regression using the GraphPad Prism software version 5.0 (San Diego, California) as described above. Selectivity indices (SI = CC_50_/EC_50_, defining the balance between cytotoxicity and antiplasmodial activity) were calculated for each test substance.

#### Data analysis

All the results represented are mean ± standard deviation (SD) from two independent experiments. Microsoft Excel Software was used to calculate the percentage of inhibition. The EC_50_ and CC_50_ values for the in vitro antiplasmodial activity were determined by non-linear regression analysis using GraphPad Prism 5.0 Software.

## Results

### Chemical characterization of the isolated natural compounds

**Fig. 3:**
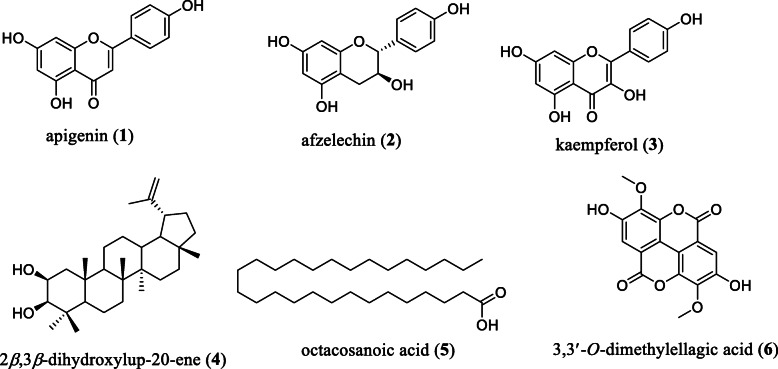
Chemical structures of compounds isolated from active fractions of the stem bark of *H. afzelii* (1–5) and *E. calophylloides* (6)

A total of 8 compounds were isolated of which compounds 3 and 5 were obtained respectively from active fractions of *H. afzelii* and *E. calophylloides* (Fig. 3).

The compounds were identified by comparing their NMR and MS data to those of previously reported compounds viz. apigenin (1) [23], afzelechin (2) [24], kaempferol (3) [25], 2*β*,3*β*-dihydroxylup-20-ene (4) [26], octacosanoic acid (5) [27] and 3,3′-*O*-dimethylellagic acid (6) [28]. Compound 6 is reported herein for the first time from *E. calophylloides*, while compounds 7 and 8 were not characterized due to their poor solubility in analytical solvents.

### Antiplasmodial activity and cytotoxicity of crude extracts from H. afzelii and E. calophylloides

The methanolic and hydroethanolic crude extracts from *H. afzelii* and *E. calophylloides* stem barks were assessed for their antiplasmodial activity against Chloroquine-sensitive 3D7 and resistant Dd2 strains of *P. falciparum* as well as for cytotoxicity activity against both *Vero* and *Raw* cells (Table 1).

**Table 1:** Extraction yield and biological activities of crude extracts

Plant species	Part	Solvent	Code	Yield (%)	EC_**50**_ (μg/mL)		RI	CC_**50**_ (μg/mL)		SI	
										***Vero***	***Raw***
					***Pf***3D7	***Pf***Dd2		***Vero***	***Raw***	***Pf***3D7 (***Pf***Dd2)	***Pf***3D7 (***Pf***Dd2)
*H. afzelii*	Stem bark	Hydroethanol	Hasb^HE^	19.50	3.32 ± 0.15	3.08 ± 0.21	0.93	> 200	> 200	> 60.24 (> 64.94)	> 60.24 (> 64.94)^a^
	Stem bark	Methanol	Hasb^M^	12.50	> 100	> 100	–	–	–	–	–
*E. calophylloides*	Stem bark	Hydroethanol	Ecsb^HE^	07.40	5.32 ± 0.03	8.72 ± 1.02	1.63	66.85 ± 4.45	31.72 ± 6.27	12.57 (7.67)	5.96 (3.63)
	Stem bark	Methanol	Ecsb^M^	08.20	7.40 ± 0.19	7.48 ± 0.07	1.01	> 200	99.09 ± 1.42	> 27.02 (> 26.74)	13.40 (13.24)^a^
Reference drugs (μM)	Artemisinin				0.014 ± 0.001	0.018 ± 0.003	1.28	NA	NA	–	–
	Chloroquine				0.018 ± 0.002	0.449 ± 0.065	24.94	NA	NA	–	–
	Podophyllotoxin				NA	NA		1.89 ± 0.38	0.76 ± 0.09	–	–

Results are expressed as Means ± standard deviation of duplicate experiments

*Hasb*^*HE*^ hydroethanolic extract of *Hymenostegia afzelii* stem bark, *Hasb*^*M*^ methanolic extract of *Hymenostegia afzelii* stem bark, *Ecsb*^*HE*^ hydroethanolic extract of *Endodesmia calophylloides* stem bark, *Ecsb*^*M*^ methanolic extract of *Endodesmia calophylloides* stem bark, *Pf Plasmodium falciparum*, *NA* not applicable, *EC*_*50*_ 50% effective concentration, *CC*_*50*_ 50% cytotoxicity concentration, *RI* resistance index, *SI* selectivity index; ^a^Crude extracts of interest

Extraction yields varied from 07.40 to 19.50% depending on the plant and the solvent of maceration. The highest extraction yield (19.50%) was obtained with the hydroethanolic stem bark extract of *H. afzelii* (Hasb^HE^) while the extraction yield was lowest (07.40%) with the hydroethanolic stem bark extract of *E. calophylloides* (Ecsb^HE^) (Table 1).

According to the recommendations of the WHO and previous published criteria, the antiplasmodial activity of natural products is categorized as follows: high (EC_50_ < 5 μg/mL), promising (5 ≤ EC_50_ < 15 μg/mL), moderate (15 ≤ EC_50_ < 50 μg/mL) and inactive (EC_50_ ≥ 50 μg/mL) [29, 30]. The hydroethanolic extract of the stem bark of *H. afzelii* (Hasb^HE^) exhibited high antiplasmodial activity against *Pf*3D7 (EC_50_ = 3.32 ± 0.15 μg/mL) and *Pf*Dd2 (EC_50_ = 3.08 ± 0.21 μg/mL). This crude extract was found to be non-cytotoxic against *Vero* and *Raw* cell lines at concentrations up to 200 μg/mL, with significant selectivity indices (SI > 60.24 and SI > 64.94) toward *P. falciparum* 3D7 and Dd2 strains. However, the methanolic extract of the stem bark of *H. afzelii* (Hasb^M^) was inactive on the assessed *Plasmodium* strains (EC_50_ > 100 μg/mL) (Table 1).

Regarding *E. calophylloides*, both hydroethanolic (Ecsb^HE^) and methanolic (Ecsb^M^) stem bark extracts showed promising antiplasmodial profiles on *Pf*3D7 (EC_50_Ecsb^HE^ = 5.32 ± 0.03 μg/mL and EC_50_Ecsb^M^ = 7.40 ± 0.19 μg/mL) and *Pf*Dd2 (EC_50_Ecsb^HE^ = 8.72 ± 1.02 μg/mL and EC_50_Ecsb^M^ = 7.48 ± 0.07 μg/mL). Moreover, both extracts (Ecsb^M^ and Ecsb^HE^) were non-cytotoxic at concentration ranging from 31.72 to > 200 μg/mL, but Ecsb^M^ presented good selectivity index (from 13.24 to > 27.02) as compared to Ecsb^HE^ (from 3.63 to 13.40) (Table 1).

Most of the resistance index values were around 1 (Table 1), suggesting equivalent potency against both chloroquine-sensitive and chloroquine-resistant strains of *P. falciparum*. Overall, the antiplasmodial and cytotoxic properties of Hasb^HE^ and Ecsb^M^ were most advantageous and they were therefore selected for fractionation.

### Anti-plasmodial and cytotoxicity profiling of fractions from the promising crude extracts

The fractionation of the hydroethanolic extract of the stem bark of *H. afzelii* (Hasb^HE^) led to 5 fractions (Fig. 1) of which 3 exhibited antiplasmodial activity ranging from promising to moderate against both chloroquine-sensitive 3D7 and resistant Dd2 strains of *P. falciparum* (Table 2). The 25% ethyl acetate-methanol fraction from Hasb^HE^ (F3) showed moderate activity against *Pf*3D7 (EC_50_ = 39.33 ± 0.00 μg/mL) and was about 3-fold more potent on the resistant *Pf*Dd2 (EC_50_ = 14.08 ± 3.17 μg/mL) with a resistance index of 0.36. The fraction was non-cytotoxic to *Vero* and *Raw* cells at concentrations up to 200 μg/mL. The 50% ethyl acetate-methanol fraction from Hasb^HE^ (F4) exerted a promising effect on *Pf*3D7 (EC_50_ = 11.85 ± 1.05 μg/mL) and a moderate activity on *Pf*Dd2 (EC_50_ = 15.11 ± 1.45 μg/mL) with a good selectivity index (SI_*Pf*3D7_ > 16.88 and SI_*Pf*Dd2_ > 13.24). Fractions F3 and F4 displayed similar potency against *Pf*Dd2 but F4 was more active against *Pf*3D7 and presented high selectivity index and higher yield compared to F3. Although the most polar fraction, methanol (F5), exhibited a moderate antiplasmodial activity, it showed unfavorable selectivity (SI_*Pf*3D7_ = 0.68 and SI_*Pf*Dd2_ = 0.54). Moreover, the antiplasmodial activity of this fraction negatively correlated with fractionation of the parent crude extract, suggesting plausible synergistic interaction between the bioactive molecules within the extract as elicitor of the observed activity.

**Table 2:** Antiplasmodial activity and cytotoxicity of fractions from selected crude extracts

Plant species	Crude extract	Fractions	EC_**50**_ (μg/mL)		RI	CC_**50**_ (μg/mL)		SI	
								***Vero***	***Raw***
			***Pf***3D7	***Pf***Dd2		***Vero***	***Raw***	***Pf***3D7 (***Pf***Dd2)	***Pf***3D7 (***Pf***Dd2)
*H. afzelii*	Hasb^HE^	F1	> 50	> 50	–	–	–	–	–
		F2	32.01 ± 9.99	> 50	–	–	–	–	–
		F3	39.33 ± 0.00	14.08 ± 3.17	0.36	> 200	> 200	> 5.09(> 14.20)	> 5.09(> 14.20)
		F4	11.85 ± 1.05	15.11 ± 1.45	1.28	> 200	> 200	> 16.88(> 13.24)	> 16.88(> 13.24)^a^
		F5	14.59 ± 0.79	18.47 ± 3.27	1.27	> 200	9.99 ± 0.49	> 13.71(> 10.83)	0.68 (0.54)
*E. calophylloides*	Ecsb^M^	F6	> 50	> 50	–	–	–	–	–
		F7	> 50	> 50	–	–	–	–	–
		F8	4.23 ± 0.20	1.98 ± 0.01	0.47	> 200	> 200	> 47.28(> 101.01)	> 47.28(> 101.01)^a^
		F9	4.77 ± 0.09	14.40 ± 0.70	3.02	> 200	> 200	> 41.93(> 13.89)	> 41.93(> 13.89)^a^
		F10	11.86 ± 0.29	26.86 ± 0.02	2.26	> 200	47.08 ± 5.29	> 16.86(> 7.45)	3.97 (1.75)

Results are expressed as means ± standard deviation of duplicate experiments

*Hasb*^*HE*^ hydroethanolic extract of *Hymenostegia afzelii* stem bark, *Ecsb*^*M*^ methanolic extract of *Endodesmia calophylloides* stem bark, *Pf Plasmodium falciparum*, *na* not applicable, *EC*_*50*_ 50% effective concentration, *CC*_*50*_ 50% cytotoxicity concentration, *RI* resistance index, *SI* selectivity index; ^a^most potent fractions

Fractionation of the methanolic crude extract of the stem bark of *E. calophylloides* (Ecsb^M^) led to 8 fractions that were subsequently pooled based on their thin layer chromatography (TLC) profiles to 5 fractions, F6[Hex-AcOEt (100:0–75:25)], F7[Hex-AcOEt (50:50–25:75)], F8(AcOEt), F9[AcOEt/MeOH (75:25)] and F10[AcOEt/MeOH (50:50–0:100)] (Fig. 2). Three out of the 5 fractions were found to possess antiplasmodial activity (Table 2). The ethyl acetate fraction (F8) presenting high antiplasmodial potency (EC_50_*Pf*3D7 = 4.23 ± 0.20 μg/mL; EC_50_*Pf*Dd2 = 1.98 ± 0.01 μg/mL) also showed no cytotoxicity against both *Vero* and *Raw* cells at concentrations up to 200 μg/mL (SI_*Pf*3D7_ > 47.28; SI_*Pf*Dd2_ > 101.01). This fraction strongly inhibited the resistant Dd2 strain about 2-fold more than the 3D7 chloroquine sensitive strain (RI = 0.47). The 25% ethyl acetate-methanol fraction (F9) demonstrated high activity against *Pf*3D7 (EC_50_ = 4.77 ± 0.09 μg/mL) and promising against *Pf*Dd2 (EC_50_ = 14.40 ± 0.70 μg/mL) and was found to be non-cytotoxic to mammalian cells at the tested concentration (200 μg/mL). The fraction F10 showed antiplasmodial activity level ranging from promising on *Pf*3D7 (EC_50_ = 11.86 ± 0.29 μg/mL) to moderate on *Pf*Dd2 (EC_50_ = 26.86 ± 0.02 μg/mL). However, F10 showed poor selectivity on *Pf*3D7 and *Pf*Dd2 with regard to Raw cells (SI_*Pf*3D7_ = 3.97; SI_*Pf*Dd2_ = 1.75). In comparison to the crude extract, the fractionation led to significant improvement of the antiplasmodial activity of F8 (Ecsb^M^: EC_50_*Pf*3D7 = 7.40 ± 0.19 μg/mL; EC_50_*Pf*Dd2 = 7.48 ± 0.07 μg/mL) whereas the activity of F10 was lower. The activity of F9 was also improved on *Pf*3D7 but decreased on *Pf*Dd2 as compared to the crude extract.

Overall, fractions F4, F8 and F9 showed higher antiplasmodial potency and selectivity. They were therefore selected for further fractionation to isolate the active ingredients.

### Anti-plasmodial and cytotoxicity of isolated compounds

The bioassay guided fractionation based on in vitro antiplasmodial and cytotoxicity tests led to the isolation of several classes of chemical constituents. Table 3 reports the biological parameters of the isolated compounds. The thresholds for the antiplasmodial activity were based on the established criteria [31] where EC_50_ < 1 μM indicates compound with excellent/potent activity; EC_50_ of 1–20 μM, good activity; EC_50_ of 20–100 μM, moderate activity; EC_50_ of 100–200 μM, low activity; and EC_50_ > 200 μM, inactive.

**Table 3:** Antiplasmodial activity and cytotoxicity of isolated compounds from selected fractions

Plant species	Fractions	Compounds	EC_**50**_ (μM)		RI	CC_**50**_ (μM)		SI	
								***Vero***	***Raw***
			***Pf***3D7	***Pf***Dd2		***Vero***	***Raw***	***Pf***3D7 (***Pf***Dd2)	***Pf***3D7 (***Pf***Dd2)
*H. afzelii*	F4	1	19.01 ± 0.72	16.39 ± 0.52	0.86	> 185.02	> 185.02	> 9.73(> 11.29)	> 9.73(> 11.29)
		2	I	I	–	–	–	–	–
		3	I	I	–	–	–	–	–
		4	I	I	–	–	–	–	–
		5^a^	NT	NT	–	NT	NT	–	–
*E. calophylloides*	F8	6	4.27 ± 0.05	1.36 ± 0.47	0.32	> 151.52	> 151.52	> 35.48(> 111.41)	> 35.48(> 111.41)
Reference drugs	Artemisinin		0.014 ± 0.001	0.018 ± 0.003	1.26	NA	NA	–	–
	Chloroquine		0.018 ± 0.002	0.449 ± 0.065	24.86	NA	NA	–	–
	Podophyllotoxin		NA	NA		4.56 ± 0.92	1.83 ± 0.22	–	–

Results are expressed as means ± standard deviation of duplicate experiments

*NT* not tested; ^a^: This compound wasn’t tested due to the quantity; *I* inactive, *NA* not applicable, *EC*_*50*_ 50% effective concentration; *CC*_*50*_ 50% cytotoxicity concentration, *RI* resistance index, *SI* selectivity index

Overall, only compounds 1 and 6 showed antiplasmodial activity with EC_50_ values of 19.01 ± 0.72 μM and 16.39 ± 0.52 μM for 1 and, 4.27 ± 0.05 μM and 1.36 ± 0.47 μM for 6, respectively against *Pf*3D7 and *Pf*Dd2, and resistance indices less than 1, indicating more pronounced potency on the resistant strain *Pf*Dd2 compared to the sensitive strain. The two compounds were highly selective with SI higher than 9. The antiplasmodial effect of compound 6, identified as 3–3′-*O*-dimethylellagic acid was 4- and 12-fold higher against *Pf*3D7 and *Pf*Dd2 respectively compared to compound 1, apigenin. More importantly, it was 3-fold more active on chloroquine resistant strain *Pf*Dd2 versus chloroquine sensitive strain *Pf*3D7 of *P. falciparum* (RI = 0.32).

As far as compound 6 is concerned, fractionation of the patent crude extract led to activity magnification by around 7 and 15 times respectively against *Pf*3D7 and *Pf*Dd2.

## Discussion

The current study aimed at identifying antiplasmodial compounds from the stem bark extracts of *H. afzelii* and *E. calophylloides*.

The hydroethanolic extract from the stem bark of *H. afzelii* (Hasb^HE^) exhibited promising antiplasmodial activity against chloroquine-sensitive *Pf*3D7 and multi-resistant *Pf*Dd2 plasmodial strains while the methanolic extract (Hasb^M^) showed no activity at up to 100 μg/mL (Table 1). This contrast in antiplasmodial activity might be explained by the compositional differences of the two extracts dragged by different solvents of extraction. To the best of our knowledge, the antiplasmodial activity of the *H. afzelii* plant extracts is being reported here for the first time. Meanwhile, studies carried out on other Caesalpiniaceae species revealed interesting antiplasmodial potencies when extracts are prepared using ethanolic and / or water. In this line, Tona and collaborators [32] reported high in vitro antiplasmodial activity (EC_50_ < 3 μg/ml) for the ethanol extract of the leaves of *Cassia occidentalis* L. Also, the aqueous extract from the fruit of *Tamarindus indica* L. showed potent activity against *P. falciparum* clinical isolates (EC_50_ = 2.042 μg/ml) [33]. The ethanolic leaves extract from *Senna occidentalis* L. inhibited the chloroquine-sensitive strain 3D7 and chloroquine-resistant strain INDO of *P. falciparum* with EC_50_ values of 48.80 μg/ml and 54.28 μg/ml, respectively [34]. Interestingly, the highly active hydroethanolic extract of *H. afzelii* (Hasb^HE^) corroborated this general trend, and additionally showed no significant cytotoxicity effect towards *Vero* and *Raw* cell lines (SI > 60). This further suggests that, Hasb^HE^ preferentially inhibited malaria parasites rather than the mammalian cells. Similar trend has previously been reported by Awantu and collaborators [12] while studying the effect of methylene chloride-methanol extracts from leaves and stem bark of *H. afzelii* on anhydrous *Artemia salina* larvae. Given that this pioneering study has unveiled high antiplasmodial potency of Hasb^HE^, species from *Hymenostegia* genus should be extensively explored for the identification of naturally occurring bioactive molecules against malaria parasites.

*Endodesmia calophylloides* use in traditional medicine for the treatment of malaria is not really documented. However, several species belonging to the Guttiferae family such as *Garcinia kola* Heckel, *Garcinia polyantha* Oliv., *Cratoxylum cochinchinense* (Lour.) Blume, *Allanblackia* spp. are widely used by traditional healers to cure malaria and related symptoms [32, 35–38]. In this work, we demonstrated that both hydroethanolic (Ecsb^HE^) and methanolic (Ecsb^M^) stem bark extracts of *E. calophylloides* exhibited promising antiplasmodial potency against both chloroquine-sensitive *Pf*3D7 and multi-resistant *Pf*Dd2 strains. These results corroborate those of Ngouamegne and collaborators who showed that hexane, ethanol and methanol crude extracts from stem bark of *E. calophylloides* exhibited potent antiplasmodial activity against the chloroquine-resistant W2 strain of *P. falciparum* with respective EC_50_ values of 9.3 ± 1.0 μg/mL; 7.4 ± 0.6 μg/mL and 12.8 ± 1.0 μg/mL [18]. These results highlighted the potential of *E. calophylloides* stem bark as source of antiplasmodial natural products with potency against sensitive and resistant strains of *P. falciparum*.

The fractionation of the promising extracts (Hasb^HE^, Ecsb^M^) led to various fractions with antiplasmodial activity (F3, F4, F5, F8, F9 and F10) (Table 2), supporting the bio-guided exploration approach. Interestingly, the results showed that fraction F8 (obtained in 100% ethyl acetate) from Ecsb^M^ extract of *E. calophylloides* was almost 2-fold more active against the multi-resistant Dd2 strain compared to the chloroquine sensitive 3D7 strain of *P. falciparum* (resistance index 0.47). This suggests the possibility of unique and novel drug targets in the resistant strain for active principles.

Compound 1, a flavonoid identified as apigenin, was isolated for the first time from *H. afzelii* stem bark by Awantu and collaborators [12]. Our findings on the antiplasmodial potency of apigenin corroborated previous reports on this compound. Indeed, Köhler and collaborators reported the antiplasmodial activity of apigenin against chloroquine sensitive *poW* and multi-resistant Dd2 strains of *P. falciparum* with EC_50_ of 19.0 μM and 28.5 μM, respectively [39]. Also, Lehane and Saliba [40] studied some common dietary flavonoids and found that apigenin was among those exhibiting antiplasmodial activity with EC_50_ values of 20 ± 3 μM against the chloroquine sensitive strain *Pf*3D7 and 13 ± 2 μM against the chloroquine resistant strain *Pf*7G8. In the same line, Vitalini and collaborators [41] showed that apigenin inhibited the growth of *P. falciparum* strains with EC_50_ of 25.4 ± 7.9 μg/mL for the chloroquine-sensitive strain D10 and 20.2 ± 6.4 μg/mL against the resistant strain W2. Amiri and collaborators [42] also demonstrated that apigenin significantly suppressed *P. berghei* parasiteamia by 69.74, 50.3, and 49.23% at concentrations of 70, 35 and 15 mg/kg/day, respectively in a murine malaria model. Muhaimin and collaborators [43] identified apigenin as major constituent in the ethanolic extract of *Macaranga gigantea* (Rchb. f. & Zoll.) Müll.Arg. leaf and incriminated it to be responsible for the antiplasmodial activity of this plant species. A phytochemical screening led Phadungrakwittaya [44] to detect high quantity of apigenin in 80% ethanolic extract of *Artemisia annua* L. leaf. Indeed, artemisinin which is nowadays the backbone of gold standard drugs for malaria management was isolated from *A. annua* L. but the identification of other components in high quantity such as apigenin in the species could support the fact that these constituents may play an additional role in the antiplasmodial properties of this plant. Studies on the putative mechanism of action of apigenin revealed that, this flavonoid act by inhibiting *P. falciparum* fatty acid biosynthesis. In fact, Tasdemir and collaborators [45] demonstrated that apigenin inhibits FabI, a crucial enzyme involved in fatty acid biosynthesis of *P. falciparum* with an EC_50_ of 50 μM. Comparing the susceptibility of both investigated malaria parasite strains to apigenin (1), it appeared that the multi-resistant strain *Pf*Dd2 was more susceptible than the chloroquine sensitive strain 3D7. These results are similar to those obtained by Vitalini and collaborators [41] who reported that apigenin was more potent against choloroquine-resistant strain *Pf*W2 (EC50 of 20.2 ± 6.4 μg/mL) as compare to the sensitive strain *Pf*D10 (EC_50_ of 25.4 ± 7.9 μg/mL). Likewise, we noticed the difference in antiplasmodial activity of isolated flavonoids, which was correlated to their chemical structures. Indeed, compound 3 (afzelechin) was inactive against both *P. falciparum* strains and differs from apigenin in that ring C does not bear a ketone function and a C=C unsaturation but has hydroxyl group on carbon 3. Thus, the ketone function and the unsaturation could play a favorable role in the antiplasmodial potential of apigenin. Foreseeable explanation could be the fact that the functional composition of ring C of flavonoids plausibly create a difference in cell permeability of the two molecules. Lehane & Saliba [40] hypothesized on the probable link between the structural difference of some flavonoids and cell permeability. In addition to the antiplasmodial activity, our findings showed that apigenin did not exhibit significant cytotoxicity effect toward *Vero* and *Raw* cell lines. This is in line with previous reports by Amiri and collaborators [42] showing that apigenin had no signs of cytotoxicity on human liver cell line Huh7 as well as membrane disruption on red blood cells. However, Matsuo and collaborators [46] reported a toxic effect of apigenin toward two normal human cells, TIG-1 and HUVE at 50% lethal concentration (LC_50_) value of 110 μM. But herein, EC_50_ of apigenin on both strains of *P. falciparum* are at least 5-fold lower than the LC_50_ values reported against both human cell lines insinuating that this compound remained selectively more toxic to the parasite than the mammalian cell lines.

Compound 6, 3,3′-*O*-dimethylellagic acid, exhibited a better antiplasmodial effect than compound 1 (apigenin) against both *P. falciparum* parasite strains and did not show significant signs of cytotoxicity. This is the first report of the antiplasmodial activity and cytotoxicity of 3,3′-*O*-dimethylellagic acid. However, 3,3′-*O*-dimethylellagic acid is a derivative of ellagic acid, a class of polyphenols well known for their antiplasmodial properties. In fact, ellagic acid and its derivatives were reported by several authors to display in vitro as well as in vivo antiplasmodial activities without toxicity [47–51]. Preliminary pharmacological target deconvolution and mechanism of action of ellagic acid and its derivatives on *P. falciparum* suggested that they act at the mature trophozoite and young schizont stages of the erythrocytic cycle of *P. falciparum* [51]. Furthermore, elaborated scientific reports have previously stated that ellagic acid derivatives could act by reducing the glutathione content and by inhibiting *β*-hematin formation inside the *Plasmodium* parasite [52, 53], leading to parasite death. Interestingly, we found that 3,3′-*O*-dimethylellagic acid was at least 7 to 15 times more active than the crude extract against *Pf*3D7 and *Pf*Dd2 respectively, validating the bio-guided approach adopted in this study. The structure-activity-relationship investigation by Sturm and collaborators [54] indicated that the number of hydroxyl groups in the ellagic acid scaffold positively correlated with the antiplasmodial activity. Indeed, authors demonstrated that two derivatives, flavellagic acid and coruleoellagic acid, obtained respectively by introducing one and two hydroxyl groups into ellagic acid polyaromatic ring system, led to more potency compared to ellagic acid. Our finding is consistent with the results of these authors given that 3,3′-*O*-dimethylellagic acid, bearing 2 times less hydroxyl groups than ellagic acid, exhibited lower potency against the same *P. falciparum* strains (*Pf*3D7 = 4.27 μM; *Pf*Dd2 = 1.36 μM) compared to the ellagic acid (*Pf*3D7 = 0.819 μM; *Pf*Dd2 = 0.351 μM).

## Conclusion

The bio-guided investigation of extracts from *E. calophylloides* and *H. afzelii* led to the isolation and identification of two known natural compounds as main antiplasmodial constituents. The antiplasmodial activity of compound 6, 3,3′-*O*-dimethylellagic acid, obtained from the methanolic extract of stem bark of *E. calophylloides* is herein reported for the first time. This compound exhibited higher bioactivity than compound 1, apigenin, isolated from the hydroethanolic extract of the stem bark of *H. afzelii*. Interestingly, both natural products were not cytotoxic on the tested mammalian cell lines at concentrations far above the median effective concentrations. The results achieved in this study support further safety profiling of the investigated plant species for their ultimate use in folk medicine as remedy against malaria. Likewise, compounds 1 and 6 have suitable bioactivity profiles, and could serve as starting points for hit optimization and hit-to-lead studies in an antimalarial drug discovery program.

## Supplementary Information

**Additional file 1. **1. NMR data of apigenin (1). 2. NMR data of afzelechin (2). 3. NMR data of kaempferol (3). 4. NMR data of 2*β*,3*β*-dihydroxylup-20-ene (4). 5. NMR data of octacosanoic acid (5). 6. NMR data of 3,3′-*O*-dimethylellagic acid (6).

## Data Availability

All data generated or analyzed during this study are included in this published article and its Additional files.

## Abbreviations

13C NMR: Carbon-13 nuclear magnetic resonance
1H NMR: Proton nuclear magnetic resonance
ACTs: Artemisinin-based combination therapies
ATCC: American type culture collection
C=C: Carbon carbon double bonds
CC_50_: 50% Cell cytotoxic concentration
CH2Cl2: Methylene chloride
DMSO: Dimethyl sulfoxide
*E. calophylloides*: *Endodesmia calophylloides*
EC_50_: 50% Effective concentration
Ecsb^HE^: Hydroethanolic extract of *Endodesmia calophylloides* stem bark
Ecsb^M^: Methanolic extract of *Endodesmia calophylloides* stem bark
EDTA: Ethylene diamine tetra acetate
EtOAc: Ethyl acetate
EtOH: Ethanol
F: Fraction
*H. afzelii*: *Hymenostegia afzelii*
H2O: Water
H2SO4: Sulfuric acid
Hasb^HE^: Hydroethanolic extract of *Hymenostegia afzelii* stem bark
Hasb^M^: Methanolic extract of *Hymenostegia afzelii* stem bark
HEPES: 2-[4-(2 hydroxyethyl)piperazin-1-yl]ethanesulfonic acid
Hex: Hexane
HNC: “Herbier National du Cameroun”
I: Inactive
LC_50_: 50% Lethal concentration
MeOH: Methanol
MHz: Mega Hertz
MS: Mass spectrometry
NA: Not applicable
NMR: Nuclear magnetic resonance spectroscopy
NT: Not tested
RI: Resistance index
RPMI: Roswell Park Memorial Institute Medium
SI: Selectivity index
UV: Ultra violet
WHO: World Health Organization
