# Supplementary material for: Identification of 3,3′-O-dimethylellagic acid and apigenin as the main antiplasmodial constituents of Endodesmia calophylloides Benth and Hymenostegia afzelii (Oliver.) Harms
Source: BMC Complement Med Ther. 2021 Jun 29;21:180. doi: 10.1186/s12906-021-03352-9 (PMC8243547; doi:10.1186/s12906-021-03352-9)
Supplement: Supplementary file 1 — Additional file 1. 1. NMR data of apigenin (1). 2. NMR data of afzelechin (2). 3. NMR data of kaempferol (3). 4. NMR data of 2β,3β-dihydroxylup-20-ene (4). 5. NMR data of octacosanoic acid (5). 6. NMR data of 3,3′-O-dimethylellagic acid (6). [file 12906_2021_3352_MOESM1_ESM.docx]

**Bio-guided fractionation identified 3,3'-*O*-dimethylellagic acid and apigenin as the main antiplasmodial constituents of *Endodesmia calophylloides*** **Benth and *Hymenostegia afzelii* (Oliver) Harms**

Rodrigue Keumoe^a,b^, Jean Garba Koffi^c^, Darline Dize^a^, Patrick Valère Tsouh Fokou^a^, Joseph Tchamgoue^c^, Lawrence Ayong^b^, Bruno Lenta Ndjakou^c^, Norbert Sewald^d^, Bathelemy Ngameni^e^, Fabrice Fekam Boyom^a*^

^a^Antimicrobial and Biocontrol Agents Unit (AmBcAU), Laboratory for Phytobiochemistry and Medicinal Plants Studies, Department of Biochemistry, Faculty of Science, University of Yaoundé I, P.O. Box 812, Yaoundé Cameroon.

^b^ Malaria Research Unit, Centre Pasteur du Cameroun, , P.O. Box 1274, Yaoundé, Cameroon

^c^Higher Teachers Training College, University of Yaoundé I, P.O Box 47, Yaounde, Cameroon

^d^Organic and Bioorganic Chemistry, Faculty of Chemistry, Bielefeld University, D-33501, Bielefeld, Germany

^e^Laboratory of Pharmacognosy and Pharmaceutical Chemistry, Faculty of Medicine and Biomedical Sciences, University of Yaounde I, P.O Box 1364, Yaounde, Cameroon.

*Corresponding authors: fabrice.boyom@fulbrightmail.org

**Content**

[**1. NMR data of apigenin (1)** 3](#_Toc40360020)

[**2. NMR data of afzelechin (2)** 5](#_Toc40360021)

[**3. NMR data of kaempferol (3)** 7](#_Toc40360022)

[**4. NMR data of 2*β*,3*β*-dihydroxylup-20-ene (4)** 9](#_Toc40360023)

[**5. NMR data of octacosanoic acid (5)** 11](#_Toc40360024)

[**6. NMR data of 3,3'-*O*-dimethylellagic acid (6)** 12](#_Toc40360025)

# **1. NMR data of apigenin (1)**


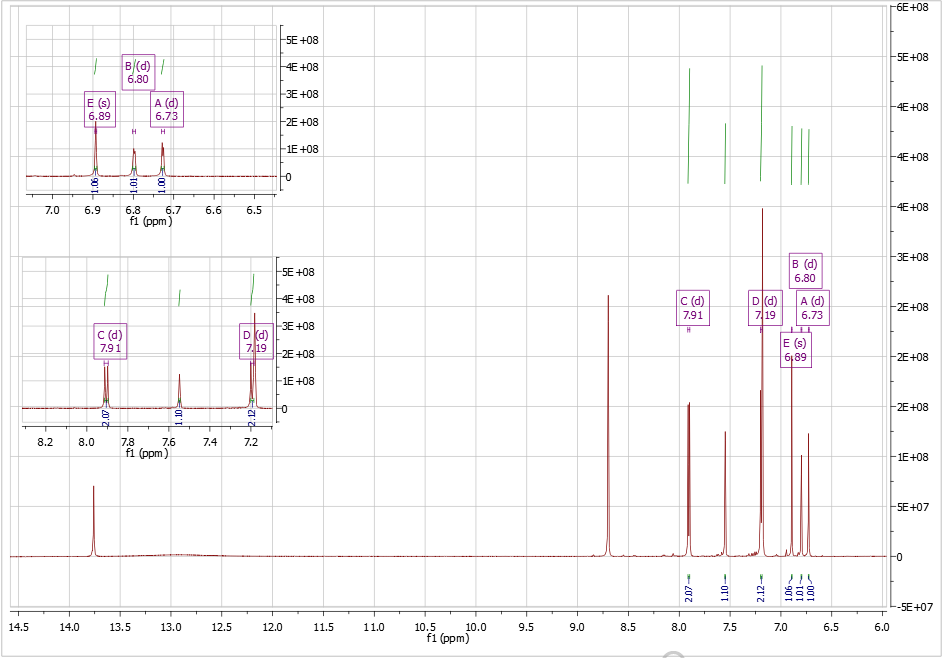


**Figure 1**: ^1^H-NMR spectrum of apigenin (**1)** (C_5_D_5_N, 500MHz, 25°C)


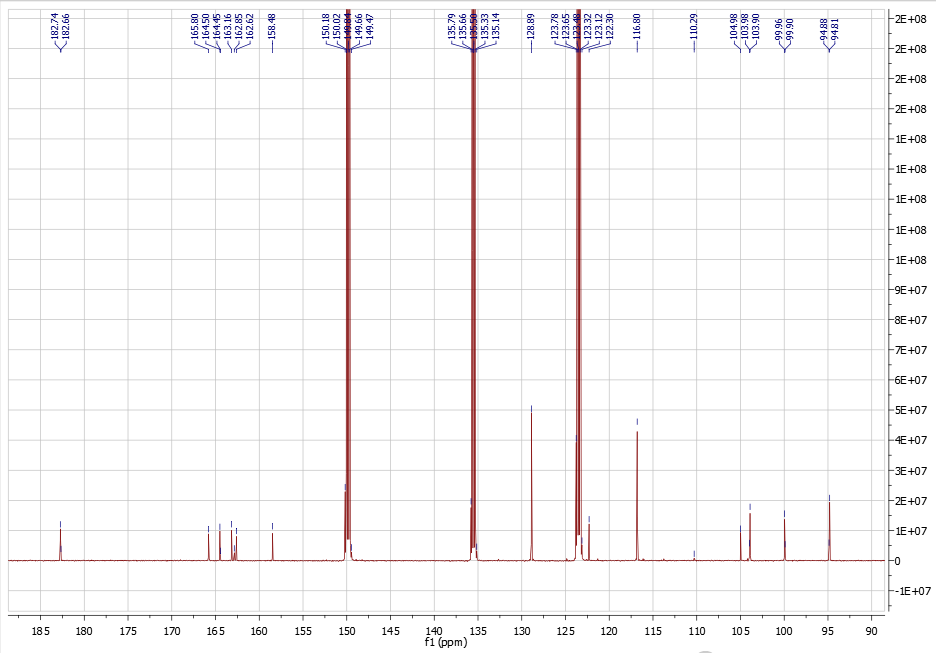


**Figure 2**: ^13^C-NMR spectrum of apigenin (**1)** (C_5_D_5_N, 125MHz, 25°C)

# **2. NMR data of afzelechin (2)**


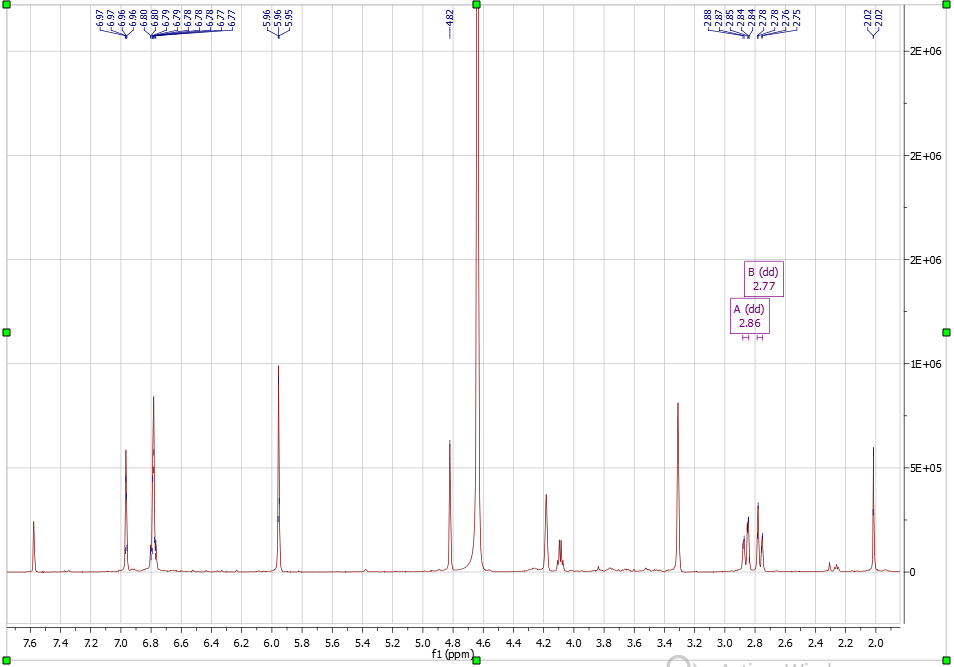


**Figure 3**: ^1^H-NMR spectrum of afzelechin (**2)** (CD_3_OD, 500MHz, 25°C)


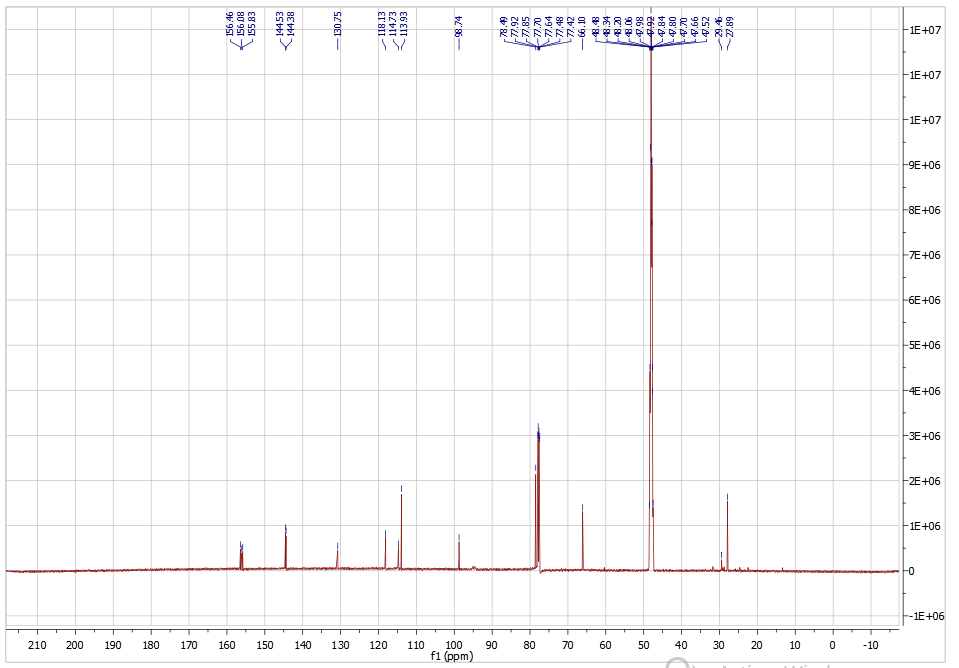


**Figure 4**: ^13^C-NMR spectrum of afzelechin (**2)** (CD_3_OD, 125MHz, 25°C)

# **3. NMR data of kaempferol (3)**


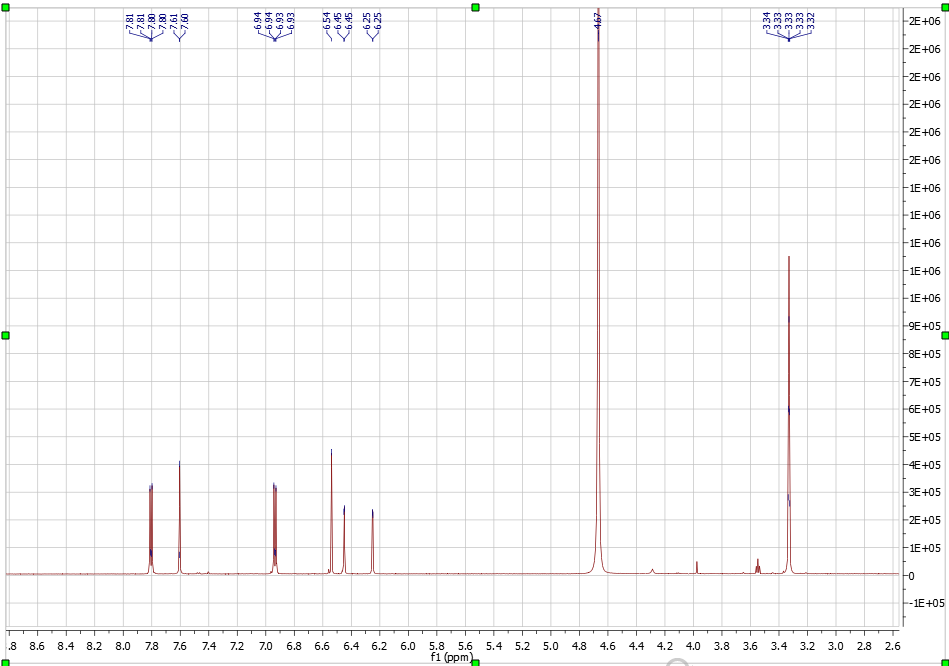
**Figure 5**: ^1^H-NMR spectrum of kaempferol (**3)** (CD_3_OD, 500MHz, 25°C)


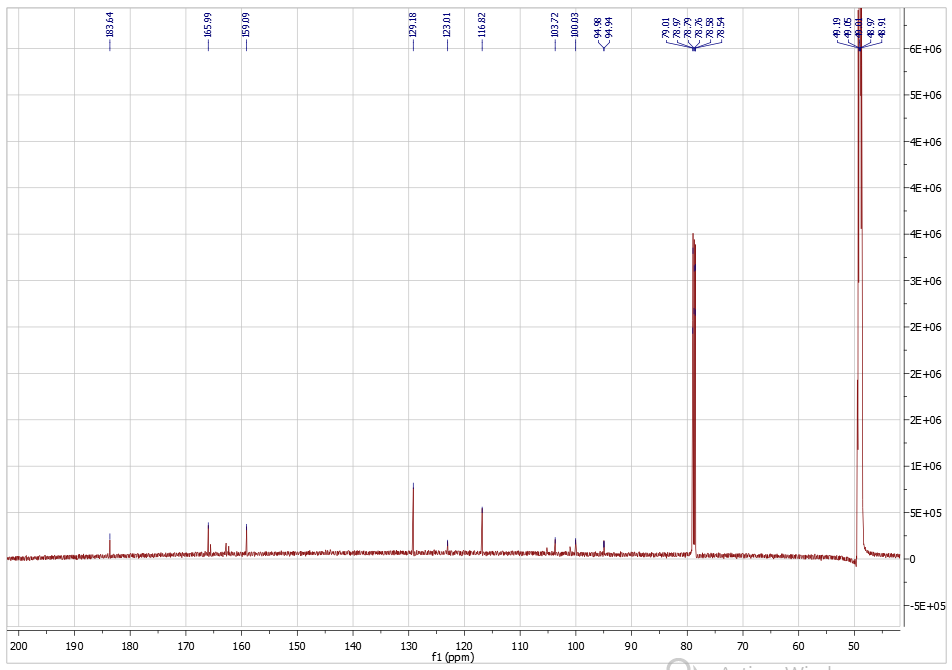


**Figure 6**: ^13^C-NMR spectrum of kaempferol (**3)** (CD_3_OD, 125MHz, 25°C)

# **4. NMR data of 2*β*,3*β*-dihydroxylup-20-ene (4)**


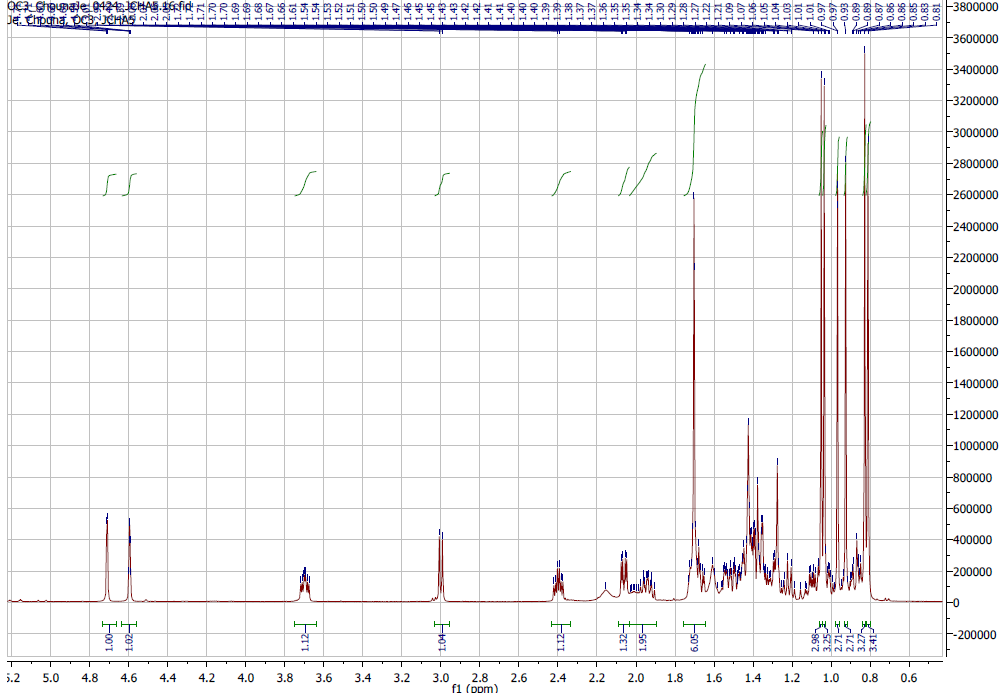


**Figure 7**: ^1^H-NMR spectrum of 2*β*,3*β*-dihydroxylup-20-ene (**4)** (CDCl_3_, 500MHz, 25°C)


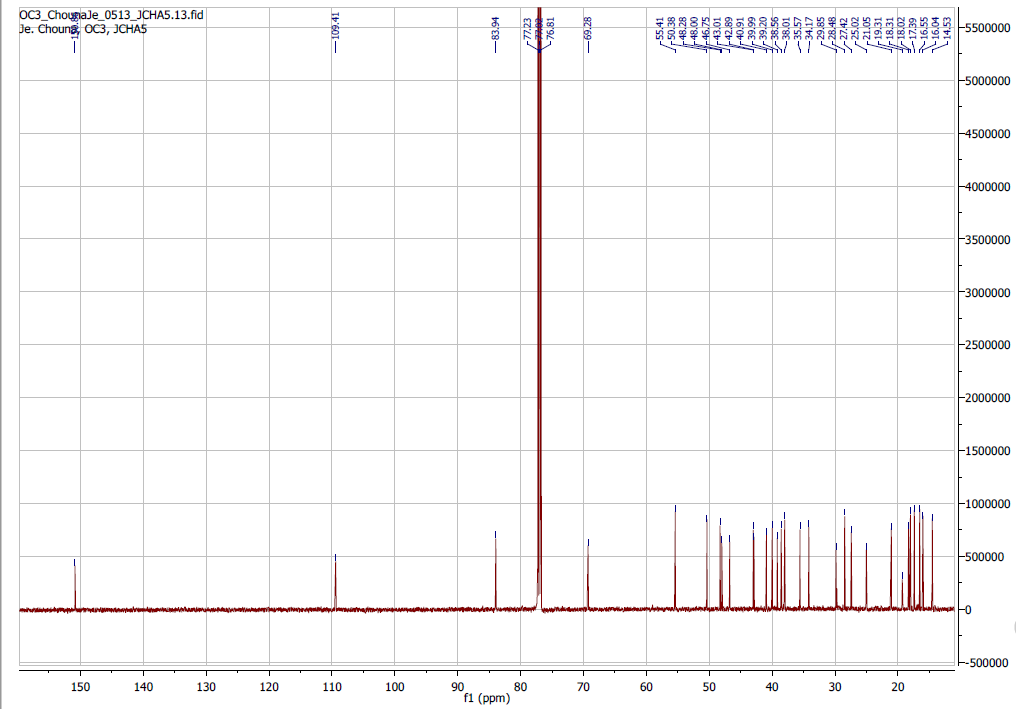


**Figure 8**: ^13^C-NMR spectrum of 2*β*,3*β*-dihydroxylup-20-ene (**4)** (CDCl_3_, 125MHz, 25°C)

# **5. NMR data of octacosanoic acid (5)**


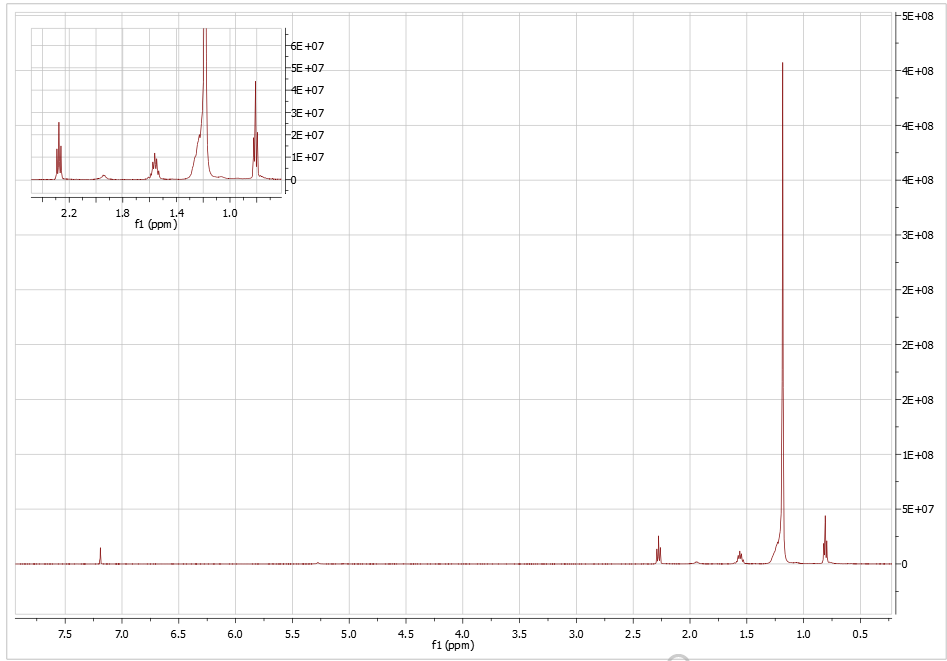


**Figure 9**: ^1^H-NMR spectrum of octacosanoic acid (**5)** (CDCl_3_, 500MHz, 25°C)

# **6. NMR data of 3,3'-*O*-dimethylellagic acid (6)**


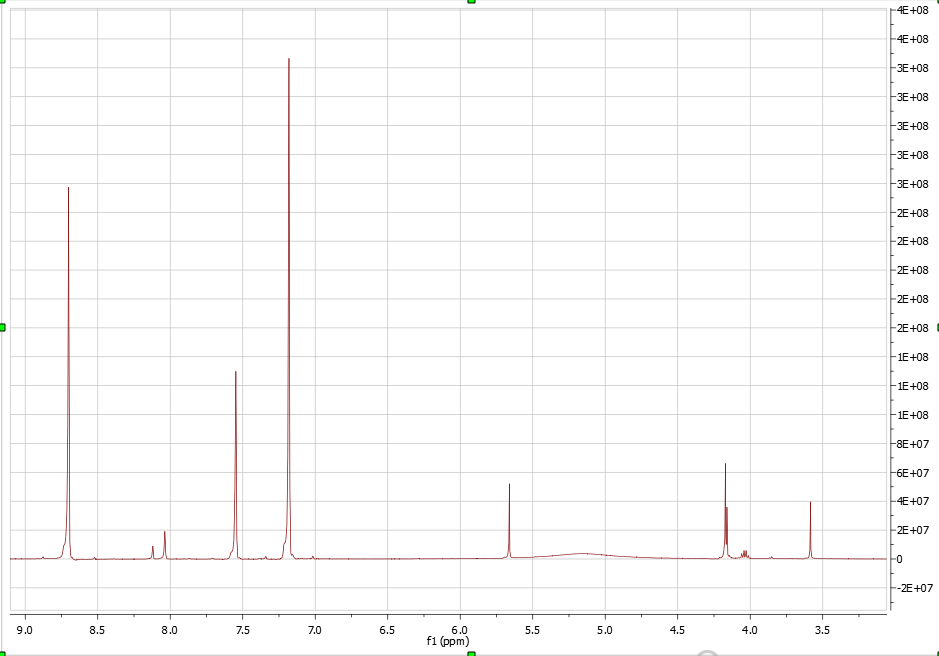


**Figure 10**: ^1^H-NMR spectrum of 3,3'-*O*-dimethylellagic acid (**6**) (C_5_D_5_N, 500MHz, 25°C)


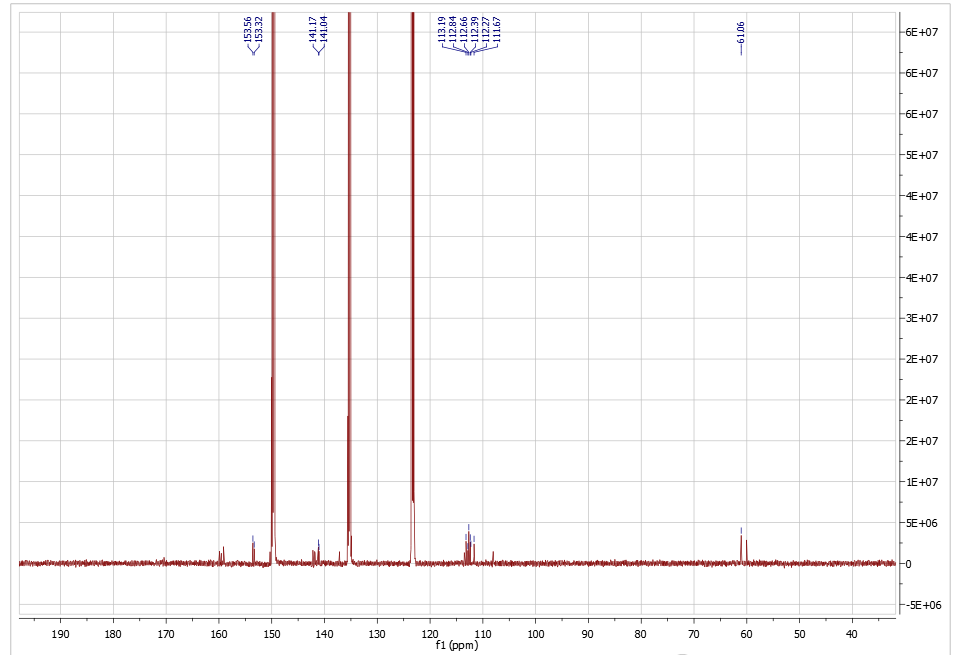


**Figure 11**: ^13^C-NMR spectrum of 3,3'-*O*-dimethylellagic acid (**6**) (C_5_D_5_N, 125MHz, 25°C)
